# Emerging Platforms for High-Efficiency Solar-Driven Interfacial Evaporation Desalination: MXene-Based Hydrogels/Aerogels

**DOI:** 10.3390/gels12020170

**Published:** 2026-02-14

**Authors:** Yue Gao, Yucheng Yang, Dongdi Yang, Fei Sun, Xiaoxiao Wang

**Affiliations:** 1 Collage of Food Science and Technology, Wuhan Business University, Wuhan 430056, China; 2 State Key Laboratory of New Textile Materials and Advanced Processing Technologies, School of Textile Science and Engineering, Wuhan Textile University, Wuhan 430200, China; 3 College of Textile Science and Engineering (International Institute of Silk), Zhejiang Sci-Tech University, Hangzhou 310018, China

**Keywords:** MXene, aerogels, hydrogels, solar-driven interfacial evaporators, desalination

## Abstract

The sustainable supply of freshwater resources is facing serious challenges due to the rapid industrial development, massive expansion of urbanization, and increasing environmental pollution. Solar-driven interfacial evaporation desalination (SIED) is considered one of the most promising candidates to tackle water scarcity and energy crisis, owing to its sustainable solar energy, abundant water sources, and pollution-free characteristic. MXene has attracted considerable attention in the domain of purified water production, owing to its remarkable properties, including tunable hydrophilicity, ease of processing, resistance to fouling, mechanical strength, and photothermal conversion capabilities. This review provides a comprehensive overview of the research progress of hydrogels/aerogels in the SIED field. Firstly, the synthesis strategy and the significantly distinctive features of MXene and its nanocomposites are outlined. Secondly, based on the photothermal conversion capacity and ease of modulation of MXene, various fabrication processes of MXene aerogels are analyzed, and the varying wettability levels of the MXene aerogel-based evaporators are discussed and summarized. Thirdly, the properties of MXene hydrogel-based evaporators are discussed from four perspectives: photothermal conversion capacity, water transport capacity, evaporation enthalpy regulation, and salt resistance. Finally, the challenges and issues related to the development of MXene hydrogels/aerogels in SIED are further discussed.

## 1. Introduction

The growing global population growth and massive expansion of urbanization, industrialization, and agricultural upgrading are exacerbating the challenge of freshwater scarcity. Additionally, the freshwater resources available for direct human use account for less than 0.36% of the total water resources [1,2,3]. Moreover, more than 1.7 billion people residing in economically backward areas do not have access to standard drinking water, and 300 million people face food insecurity due to water scarcity [4,5]. In order to settle these conundrums, some tactics and strategies are continuously put forward to produce fresh water, mainly embracing ion exchange, multi-stage flash evaporation, reverse osmosis membrane, electrodialysis, distillation, etc. [6,7]. However, these technologies conventionally require complicated and costly apparatus and consume fossil fuels, which can generate large amounts of greenhouse gases and pollute the environment [8,9,10]. Therefore, it is essential to develop a highly efficient and low-budget water purification technology with low energy consumption to solve this dilemma.

Because of pollution-free and sustainable characteristics and the large total energy reserves, solar energy has attracted significant interest from researchers [11,12]. Currently, the exploitation of solar energy to desalinate seawater can not only diminish pollution from fossil fuel combustion but also supply sustainable access to freshwater resources [13,14,15]. Due to its features of environmental friendliness, renewable nature, and economic efficiency, solar-driven interfacial evaporation seawater desalination (SIED), as one of the solar steam generation technologies, has gained wide attention in recent years [16,17]. The improvement of solar interfacial evaporation technology is mainly concentrated on the component design of photothermal conversion materials and structural architecture engineering. The admirable photothermal materials are essential to possess unique features such as high light absorption, low enthalpy of evaporation, low thermal conductivity, suitable pore space, etc. [18,19]. Gu et al. [20] developed a lignin/wood-based solar evaporator, which had a photothermal conversion capacity as high as 91.74%, occupied the low evaporation enthalpy of water, and maintained the original pore structures of natural wood.

Transition-metal carbides, nitrides, and carbonitrides, denoted by MXene, are a class of two-dimensional materials with unique photothermal conversion properties that have recently emerged as ideal photothermal materials for desalination domains [21,22,23]. And MXenes have featured a variety of exceptional properties, including high electrical conductivity, favorable flexibility, defined surface groups, adjustable hydrophilicity, outstanding mechanical strength, etc. [24,25]. To date, more than 40 MXenes have been successfully synthesized, comprising Ti_3_C_2_T_x_, Ti_2_CT_x_, Nb_4_C_3_T_x_, Ti_3_CNT_x_, Ta_4_C_3_T_x_, Nb_2_CT_x_, V_2_CT_x_, and Nb_4_C_3_T_x_; however, there have been more theoretical predictions based on the available MAX phase precursors [26,27]. Among these, titanium-based MXenes, for instance, Ti_3_C_2_T_x_ and Ti_2_CT_x_, are the most promising candidates for in the field of SIED due to their elemental abundance and non-toxic decomposition products [28,29]. In particular, Ti_3_C_2_T_x_ is the most studied MXene. For example, Liu et al. [30] achieved integrated hydrothermal management by establishing a hierarchical Ti_3_C_2_T_x_ MXene-reduced graphene oxide (rGO) sponge with anisotropic thermal conductivity and axial-directional water transport channels. The axial-directional frame provides prompt water transport channels to the air–water interface; meanwhile, the vertically oriented MXene nanosheets make the thermal conductivity of MXene-rGO composite in the radial direction much higher than the axial thermal conductivity, thus inhibiting heat loss. Inspired by nature, Lei et al. [31] ingeniously designed and successfully wove a three-dimensional (3D) honeycomb-like fabric adorned with Ti_3_C_2_T_x_. The honeycomb architecture with intermittent concave clusters produced the optimum light-trapping through multiple scattering and omni-directional light absorption, which operated synergistically with the light absorbance of MXene. And the hierarchical MXene–rGO sponges exhibited an evaporation rate of 2.35 kg m^−2^ h^−1^ under 1 sun and maintained 85% energy efficiency under weak sunlight (0.5-sun).

**Figure 1:**
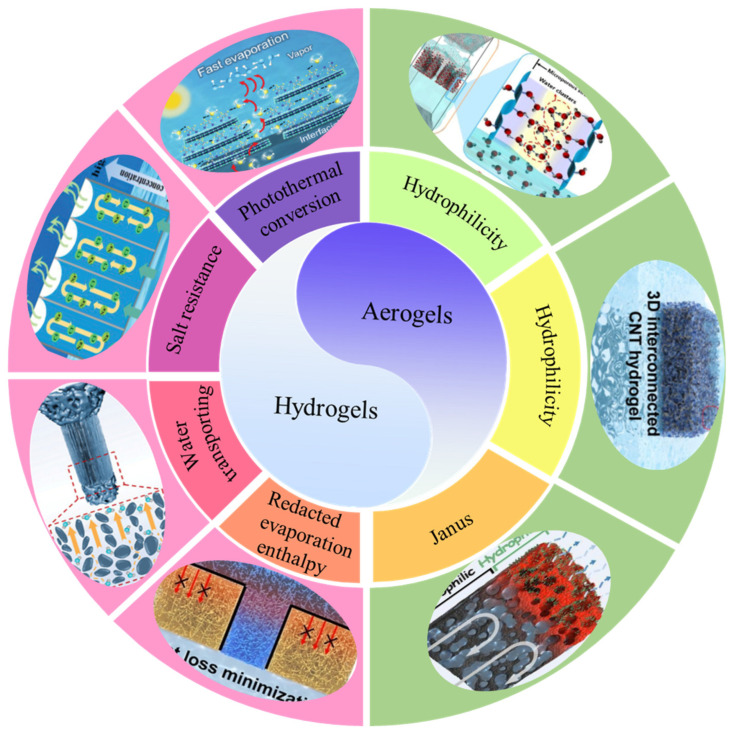
MXene-based aerogels/hydrogels for solar-driven interfacial evaporation desalination.

In the last few years, various MXene gel composites (hydrogels and aerogels) with configurations have been widely designed for use as solar-driven interfacial evaporators. In this context, it is timely to summarize and analyze the recent progress in hydrogel and aerogel solar-driven interfacial evaporators in an attempt to provide better design guidance for the future development trend (Figure 1). This review is organized into four major components. Firstly, we systematically introduce and describe the preparation process and significantly distinctive features of MXene. Secondly, various fabrication processes of MXene aerogels are analyzed, and the hydrophilicity, hydrophobicity, and distinctive asymmetric wettability of the MXene aerogel-based solar-driven interfacial evaporators are discussed and summarized. Then, the design principles to enhance water evaporation efficiency of the hydrogel-based solar-driven interfacial evaporators are outlined, primarily encompassing the photothermal management of interfaces, highly interconnected water transport channels, reduction of evaporation enthalpy, and salt resistance. Finally, the future development directions and challenges are proposed.

## 2. Structure, Synthesis, and Characteristics of MXene

### 2.1. Preparation of MXene Nanosheets

**Figure 2:**
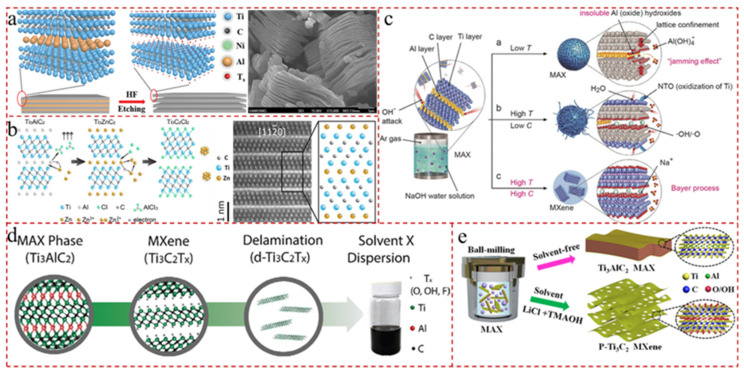
(**a**) Schematic illustration for the preparation of MXene and MWCNTs and SEM images of initial MXene particles after HF etching [34]. (**b**) A general approach to synthesizing a series of Zn-based MAX phases and Cl-terminated MXenes, and a high-resolution STEM image of Ti_3_ZnC_2_ [38]. (**c**) The reaction between Ti_3_AlC_2_ and NaOH water solution under different conditions [36]. (**d**) Schematic describing the synthesis and dispersion of Ti_3_C_2_T_x_ in organic solvent X [37]. (**e**) Schematic illustration of the preparation of P-Ti_3_C_2_ by chemical-combined ball milling in TMAOH and LiCl solvent [39].

MXene materials are transition metal carbides, nitrides, and carbonitrides with the general chemical formula M_n+1_X_n_ (or M_n+1_X_n_T_x_), where M stands for early transition metals (e.g., Ti, Sc, Nb, Mo, Ta, and V), X is carbon and/or nitrogen, and T indicates surface functional layers added by etching (e.g., -O, -F, or -OH) [32,33]. The two fundamental tactics of preparation are top-down approach and bottom-up approach. The former approach describes selective etching of the A-layer from a series of MAX-phase precursors to yield MXene and mainly comprises conventional HF etching [34], acid/salt-based HF etching [35], fluorine free strategy [36], organic solvents etching [37], uniform surface terminations [38], acoustic method [39], etc., as shown in Figure 2. For example, Sarycheva et al. [40] utilized HF as an etching agent to obtain large multilayered Ti_3_C_2_ particles. In order to evade low surface area and inhomogeneous edge terminations generated by hazardous HF etching, Zhang et al. [41] adopted the NaCl/ZnCl_2_ salt, as the phase transition material, to synthesize Cl-terminated MXene (Ti_3_C_2_Cl_2_) with tunable in-plane porosity via a eutectic mixture etching strategy. The obtained MXene possessed substantial increment in material mesoporosity and a fourfold increase in surface area. Importantly, various synthesis processes provide different surface chemistry, morphology, and intrinsic properties, which can in turn have a profound effect on the electrochemical properties and activity of MXene. Therefore, it is imperative to select the most appropriate method based on the specific prerequisite. Although most MXenes with standard accordion-like layer structures are still prepared by chemical etching with fluorine-containing solutions, fluorine-free solution etching methods are booming.

Bottom-up is a direct manner of manufacturing MXene by depositing elements onto the substrate via atomic layer deposition or chemical vapor deposition (CVD). As early as 2004, Emmerlich et al. [42] used direct-current magnetron sputtering to deposit Ti, Al, and C elemental targets onto substrates to prepare Ti_3_AlC_2_ MAX membranes. Öper et al. [43] grew thin and large-area Mo_2_C flakes in a controlled manner by using CVD, avoiding surface functionalization and limited lateral dimensions. Moreover, the thickness of Mo_2_C flakes was varied from 7 to 145 nm, and the substrate surface coverage was increased from 11% to 68% via controlling the flow rate of CH_4_, the catalyst/precursor (Cu/Mo) ratio, and the carrier gas (N_2_/H_2_) ratio. In addition, MXene surfaces prepared by this method lack surface termination compared to etching for certain applications. Wang et al. [44] demonstrated a direct synthetic route for the scalable and atom-economic synthesis of MXene through the high-temperature reaction between Ti, graphite, and TiCl_4_. The CVD method has the potential to produce high-quality 2D MXene with large lateral dimensions and few defects. However, CVD is not suitable for the fabrication of large-area films, and only a few types of carbide MXenes are produced by CVD.

### 2.2. Properties of MXene Nanosheets

**Figure 3:**
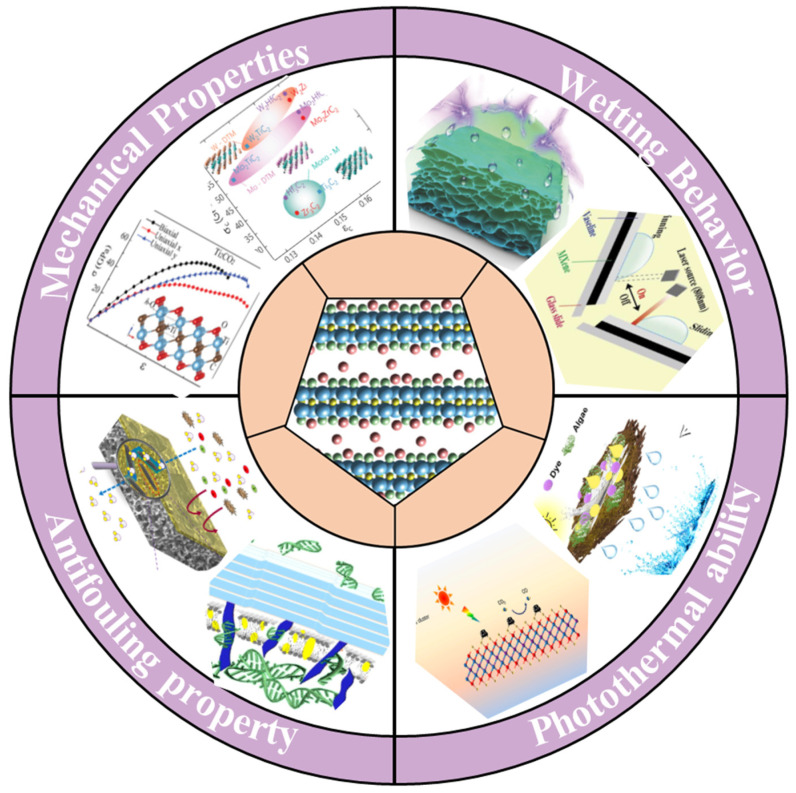
The main properties of the MXene composite evaporator.

Among various two-dimensional materials, such as graphene oxide (GO), CNTs, transition metal dichalcogenides (TMDs), metal–organic frameworks (MOFs), and covalent–organic frameworks, MXene nanosheets possess several unique advantages as fundamental materials for solar-driven evaporators. This section briefly reviews the tunable properties of MXene for seawater desalination, including mechanical properties, wetting behavior, photothermal ability, antifouling property, etc. (Figure 3).

#### 2.2.1. Mechanical Properties

**Figure 4:**
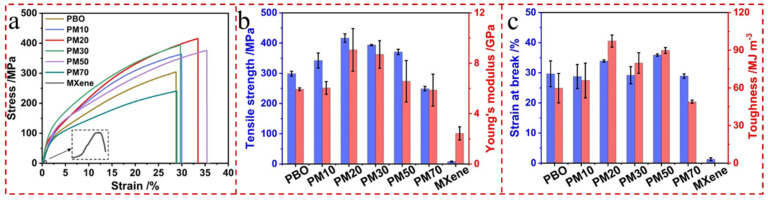
(**a**) Typical stress—strain curves of PBO/MXene films with different MXene contents [50]. (**b**) Tensile strength and Young’s modulus [50]. (**c**) Strain at break and toughness [50].

Considering the practical application requirements of solar-driven interfacial evaporator systems, 2D MXene with excellent mechanical properties can easily meet the long-life endurance and durability. Due to its high Young’s modulus of up to approximately 0.33 TPa, monolayer Ti_3_C_2_T_x_ MXene has higher elasticity than GO and other solution-treated similar 2D materials [45,46]. The electrostatic repulsion between MXene nanosheets and their excellent hydrophilicity enable uniform dispersion of MXene nanosheets within the hydrogel matrix [47]. This provides numerous rigid physical crosslinking sites, with polymer chains tightly entangled around these MXene nanosheets via hydrogen bonding between surface functional groups. Remarkably, MXene nanosheets’ functional groups (e.g., -F, -O, -OH, etc.) can be easily and firmly bonded with polymers to form the secondary crosslinked networks. These polymers primarily encompass hexadecyltrimethylammonium bromide, polyethyleneimine, poly(dimethylsiloxane) (PDMS), poly(ethylene oxide) (PEO), PVA, etc. This significantly improves the mechanical properties of the acquired MXene composite hydrogel materials [48,49]. Among them, it is worth emphasizing that PDMS (hydrophobic) and PVA (hydrophilic) are conventional aerogels with dissimilar wettability behaviors. For instance, Liu et al. [50] prepared a nacre-inspired high-performance poly(p-phenylene-2,6-benzisoxazole) (PBO)/MXene nanocomposite membrane by a sol–gel film conversion method with a homogeneous gelation process. And the PBO/MXene-20% nanocomposite film achieved the optimal mechanical property values, including a tensile strength of 416.7 MPa, a Young’s modulus of 9.1 GPa, and a toughness of 97.3 MJ m^−3^, which were 1.40, 1.54, and 1.63 times higher than those of pure PBO film, respectively (Figure 4a–c).

#### 2.2.2. Wetting Behavior

Fundamentally, the wetting behavior of liquid droplets on MXene surface is associated with the cooperation between the solid and liquid phases, and such cooperation is further dependent on several factors, comprising electrostatic interactions, van der Waals forces, and hydrogen bonding [51,52]. Owing to its surface rich in functional groups (-F, -O, -OH, and -Cl), MXene exhibits exceptional hydrophilicity. It is widely recognized that the diverse functional groups collectively determine MXene’s hydrophilic properties [53,54]. The hydrophilicity of MXene can be further regulated by varying the category and number of surface terminations. The hydrophilicity and hydrophobicity of solar-driven interfacial evaporators have a great impact on their performance and application [55]. For example, hydrophobic evaporators exhibit more superior self-cleaning capability and outstanding salt tolerance than hydrophilic evaporators, while hydrophilic evaporators display more eminent water transport capacity. The introduction of MXene with hydrophilic surface groups is a simpler and more robust method of improving wetting behavior than surface grafting and coating methods. Ma et al. [56] utilized classic molecular dynamics simulation to systematically explore the influences of termination group, comprising fluorine (F), oxygen (O), and hydroxyl groups (OH), on the desalination capability of Ti_3_C_2_T_x_ membrane. And the water permeability through MXene channel with various surface terminations followed the order of F > O > OH.

#### 2.2.3. Photothermal Ability

Thermally driven membrane distillation including solar evaporation is one of the most promising candidates for purifying seawater. Owing to their pronounced absorption peaks in the near-infrared region, which resemble those of nanoparticles, the photothermal conversion mechanism of MXene falls under the localized surface plasmon resonance (LSPR) effect [57,58,59]. Due to their exceptionally high metallic conductivity, when illuminated, MXene nanosheets exhibit surface plasmon resonance (SPR) at their surfaces as free electrons resonate with incident photons, confining this resonance within a finite area. Upon exposure to light within specific wavelength regions, surface electrons resonate with photons. Excited-state electrons transition from occupied to vacant states, forming hot electrons [60,61]. During the energy decay process, a portion of this energy is transferred to the lattice via excited phonons, causing a rapid localized temperature increase within the system. And MXene’s photothermal conversion is driven by electron excitation, forming and subsequently relaxing electron–hole pairs. When photon energy equals or exceeds the bandgap energy, electrons transition from the valence band to the conduction band. During non-radiative relaxation, these excited electrons release energy in the form of phonons (thermal energy), generating local lattice heating [62,63]. Moreover, MXene exhibits excellent broadband absorption, whereby incident photons traverse the lattice structure and undergo internal reflection between layers, thereby triggering substantial photon absorption [64,65,66]. Due to the unique nanostructure feature, broad solar-spectrum absorption, and intriguing photothermal effects, MXene has been extensively applied in the field of seawater desalination [67,68]. The metallic properties and local surface plasmon resonance bestow MXene with eminent photothermal conversion and photocatalytic activities [69,70]. Although MXene has many merits as an evaporator, pure MXene illustrates limited evaporation efficiency and rate. Some functional materials have been combined with MXene to tackle these challenges. For example, Wang et al. [71] took advantage of polydopamine-modified MXene as a photothermal material, and the honeycomb porous structure unidirectionally aligned microchannels and endowed the biomimetic aerogel with a high water evaporation rate and energy efficiency (93.6%) under 1 sun.

#### 2.2.4. Antifouling Property

Another compelling norm for aerogels/hydrogels in water purification is their antifouling resistance against salts, organics and microorganisms. The antifouling MXene-based aerogels/hydrogels avert frequent replacement, therefore broadening their service life and saving costs [72]. Long et al. [73] proposed a carboxyl-modified MXene (MCOOH)-CaCO_3_/TA/SA(CaTS) bilayer composite hydrogel, and the as-prepared MCOOH-CaTS composite hydrogel had excellent separation performance for a wide range of emulsions, maintaining high levels (3979.5 L m^−2^ h^−1^ bar^−1^ and 99.96%) over five continuous separation cycles. The MCOOH-CaTS composite hydrogel can achieve 92.5% antimicrobial properties in the treatment of actual oily wastewater. Notably, the hydrophilicity of the MXene nanosheets facilitates the self-cleaning of MXene against oil or other organic contaminants, which is conducive to the development of MXene materials for water purification [74,75]. Zeng et al. [76] inserted titanium oxide nanotubes (TiONT) into the interlayer space of MXene adorned with polyethersulfone bilayer membrane. The retention rate of the emulsion outweighed 99.7% for the obtained polyethersulfone-supported MXene/TiONT (PMT) membrane, and it possessed excellent antifouling and self-cleaning properties. The flux recovery rates of the bovine serum albumin- and humic acid-fouled PMT membranes jumped to 83% after 2 h and 100% after 1 h, respectively, under visible light irradiation.

#### 2.2.5. Stability

It is imperative to exercise control over the chemical stability of MXene, as this is instrumental in determining the performance of solution processing and the eventual properties of the final membrane [77,78]. In the presence of the inert gas atmosphere, Ti_3_C_2_T_x_ (T_x_ = F or OH) MXene remains stable below 800 °C and exhibits only trace oxidation (forming anatase) around 500 °C. However, exposed metallic atoms on the MXene surface are more prone to oxidation, potentially triggering spontaneous oxidation reactions [28,66]. The storage of colloidal MXene in an argon-filled vessel at low temperatures has been demonstrated to extend its shelf life. It is noteworthy that MXene exhibits significantly enhanced stability in dried forms (e.g., films) and when incorporated within polymer matrices (e.g., PVA). Moreover, annealing MXene (Ti_3_C_2_) in a hydrogen atmosphere substantially improves its stability [35,48].

## 3. MXene-Based Aerogel

MXenes exhibit distinctive metallic properties in the presence of metallic-like free electrons, demonstrating unique LSPR effects that confer strong light absorption capabilities and exceptional photothermal conversion performance. They exhibit high absorption sensitivity across an ultra-broad wavelength range of 300–2500 nm and achieve nearly 100% intrinsic photothermal conversion efficiency. Furthermore, the presence of surface functional groups such as -OH, -F, and -O endows MXenes with abundant hydrophilic two-dimensional interlayer water channels, ensuring ample water transport pathways. These properties confer promising potential for application in solar-driven evaporation systems.

### 3.1. Preparation of Aerogels

The manufacturing process of aerogels incorporates the following three stages: (1) preparation of suspension; (2) gelation; and (3) replacement of solvents with air by drying, while maintaining its three-dimensional porous structure. Among them, the third phase is the most crucial, as it has a very critical impact on the microstructures of aerogels, mainly encompassing atmospheric-drying, freeze-drying, and supercritical-drying methods [79,80].

**Figure 5:**
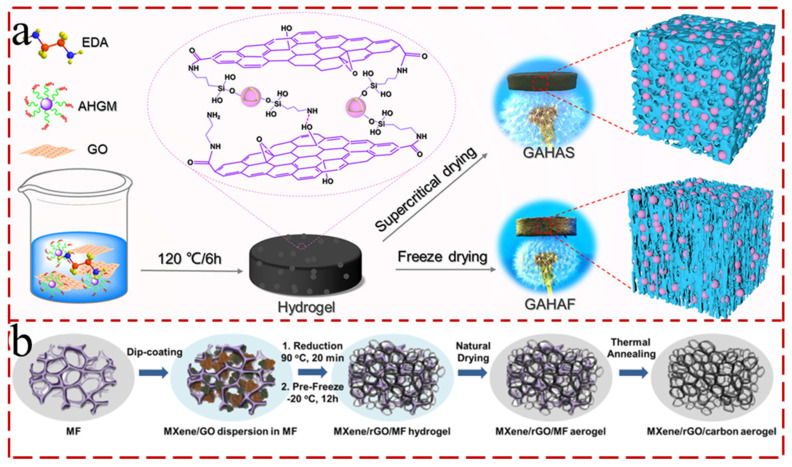
(**a**) Schematic illustration of the preparation process and structures of GAHAF and GAHAS [85]. (**b**) Schematic illustration of procedures in preparing naturally dried MGC aerogels [83].

The ambient-pressure drying is carried out using solvents with low surface tension, which can cause shrinkage of the pores at the solid–liquid–vapor interface. Such phenomena are usually averted by chemically treating the surface of the pore wall with non-polar groups to inhibit further compression by virtue of surface charges [81,82]. And silylation, as a surface modifier, is utilized to realize the surface passivation of gels, therefore averting the contraction of active groups on the pore walls during drying, and bringing about higher porosity. Jiang et al. [83] ingeniously developed MXene/rGO/carbon (MGC) aerogels by employing porous melamine foam as the robust template, thereby preventing volume shrinkage during the ambient-pressure drying process (Figure 5b). The MGC-50 aerogel exhibited an outstanding evaporation rate of up to 1.48 kg m^−2^ h^−1^ under 1 sun illumination. However, during the atmospheric-pressure drying process, the liquid surface tension inevitably generated damaged network frameworks and impaired the capillary forces of aerogels, giving rise to macromorphological collapse.

The supercritical-drying strategy is the earliest large-scale fabrication of aerogels by transforming the drying medium into the supercritical state under high temperature and pressure, which has not undergone any phase change and avoids the surface tension generated by the liquid pulling on the solid structure [74,84]. Wang et al. [85] synthesized a novel aerogel composite consisting of rGO and 3-aminopropyltriethoxysilane-modified hollow glass microspheres as high-efficiency solar steam generation by supercritical CO_2_ drying. RGO conferred the aerogel composite superhydrophilic wettability, high light absorption (93%), and excellent photothermal conversion efficiency of 89.13% under 1 sun (Figure 5a). There are no capillary forces in the supercritical-drying method, and the aerogel cannot shrink or undergo structural damage [86]. However, the inherent defects of the approach, involving a complex process, dangerous operation, high cost, etc., extremely restrain the large-scale production of aerogels.

Among the three drying methods, freeze-drying is the most widely used, as it can diminish capillary forces by mitigating discrepancies in the gas–liquid phase and maintain dimensional stability of aerogels over drying [87,88]. The freeze-drying process involves two stages: the sol–gel solution initially converts into ice at the freezing point, and the obtained ice crystals subsequently sublimate to maintain the porous 3D architectures. In addition, the pore dimension distribution of the aerogels can be further oriented by adjusting the growth rate and direction of the ice crystals [89,90]. For example, Pan et al. [91] developed PVA/cellulose nanofibers (CNFs)/MXene aerogels via glutaraldehyde acetal crosslinking reaction and directional freeze-drying technology. The vertically porous structure and lateral evaporation of PVA/CNFs/MXene aerogels can significantly enhance solar absorption, thermal positioning, moisture transport, and salt tolerance. Due to the reduced enthalpy of water evaporation (1696 kJ kg^−1^), the PVA/CNFs/MXene aerogel exhibits an excellent evaporation rate (4.30 kg m^−2^ h^−1^) and high solar steam efficiency (164.3%).

The disordered internal structure of aerogels gives rise to randomly distributed water transport pathways, thereby hindering connectivity with bulk water reservoirs. This has the effect of impeding the sustained water supply to the evaporator surface, whilst also inducing salt deposition [77,92]. Conversely, this significantly compromises the device’s operational efficiency. Drawing inspiration from plant transport systems, whose fiber bundles with vertical channels and hierarchical pore structures enable bottom-up water transport and top-down delivery of photosynthesized nutrients, provides an ideal model for solar evaporators [93,94]. Vertically oriented aerogels obtained via directional freeze-drying exhibit reversible compressibility and longitudinal water transport capability [95,96]. Although directional freezing and customized templates optimize evaporator microstructure and enhance evaporation efficiency, metal thermal molds and extreme temperature differentials complicate fabrication. Channels formed by directional freezing exhibit randomness and prove difficult to control flexibly. Furthermore, evaporators with hierarchical porosity structures efficiently transport moisture while reducing salt accumulation, thereby significantly enhancing evaporation efficiency [97,98]. However, single millimeter-scale macropores cannot provide robust capillary forces, and insufficient hydrophilicity limits water transport capacity. Consequently, the fabrication of evaporators that combine high evaporation efficiency, controllable structure, and practicality remains challenging.

### 3.2. Functional MXene-Based Aerogels

**Figure 6:**
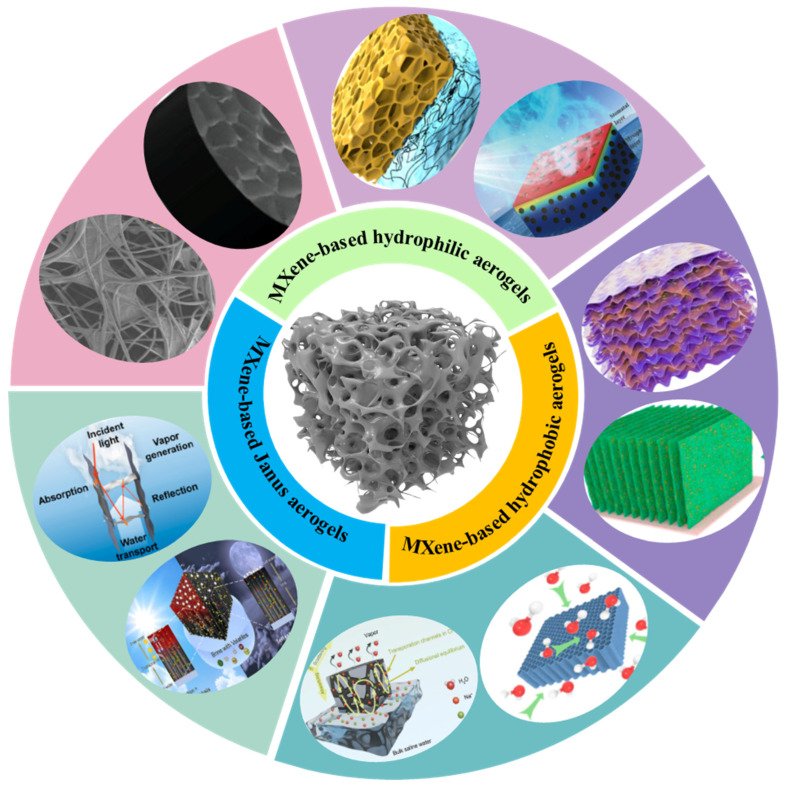
MXene-based aerogels for solar-driven interfacial evaporation desalination.

Aerogels have been an integral branch of nanomaterial research, and MXene has obtained adequate attention by virtue of its unique surface chemical structure and special physicochemical properties [99,100]. Due to their higher elasticity modulus than GO, convenient structural design, and outstanding compatibility with polymer components, various MXene composites with various dimensions (0D, 1D, 2D, and 3D) have attracted extensive interest in water treatment; electromagnetic interference shielding; energy harvesting, storage, and conversion; etc. [101,102]. Among them, MXene-based aerogels have emerged in solar-driven interfacial evaporation desalination (SIED) and display outstanding performance. The hydrophilicity/hydrophobicity of MXene has an important impact on the performance of MXene-based aerogels for SIED, and it accelerates the evaporation property by adjusting the wettability of the solar evaporator surface. Therefore, this section will concentrate on the performance of MXene-based aerogels (hydrophilic, hydrophobic, and Janus) used in solar evaporators (Figure 6).

#### 3.2.1. MXene-Based Hydrophilic Aerogels

The evaporation rate and efficiency of MXene is associated with the hydrophilic properties of MXene-based aerogels for continuous water transfer and vapor escape [103]. Inspired by bio-materials, hydrophilic materials which are based on high surface energy and surface roughness have become a hot research topic. And the hydrophilicity of MXene composites can be modulated by constructing 3D-structure aerogels consisting of crosslinking agent components and introducing functional materials. Here, MXene-based hydrophilic aerogels are primarily subdivided into nanofiber (nanowire)–MXene aerogels and 2D carbon–MXene composite aerogels.

The nanofibers (nanowires) containing hydrophilic groups that are introduced into the MXene aerogels can not only impart hydrophilicity to the surface of the aerogels but also improve the mechanical properties, resilience, and reusability of the aerogels. And the nanofibers (nanowires) are able to endow the composite aerogels with abundant channels for water transfer and vapor escape. Singh et al. [104] developed advanced composite aerogels (MX@ANF aerogels) with a water contact angle (WCA) of 52° as solar evaporators. This was achieved by combining MXene and aramid nanofibers. The MXene facilitated light absorption and thermal conversion capabilities (solar steam efficiency of 93.8% under 1 sun). Meanwhile, the aramid nanofibers also augmented water transport (1.48 kg m^−2^ h^−1^ under 1 sun) through enhanced hydrophilicity. The unique localized surface plasma resonance effect makes Ag nanowires (Ag NWs) special light absorbers, and the insertion of Ag NWs between the MXene layers can avoid layer stacking. Tao et al. [105] developed Ag nanowires/MXene/aramid nanofibers (Ag/MXene/ANFs) composite aerogel photothermal evaporators with 3D micropores and layered structures. The Ag/MXene/ANFs composite aerogels possessed excellent mechanical properties, abundant water transport channels, excellent hydrophilicity (WCA of 0°), and reducing heat loss with an evaporation rate of 2.21 kg m^−2^ h^−1^ with an energy efficiency of 92% under 1 sun. Biomass nanofibers possess the cheap, easily available, environmentally friendly, favorably hydrophilic, and non-polluting features necessary for efficient solar interface evaporation. Chen et al. [106] fabricated a kapok fiber/MXene (KFs–MXene) aerogel as a solar steam generator, in which the unique hollow structures of kapok fibers facilitated water transport and granted superhydrophilic properties, and MXene endowed the KFs–MXene aerogel with prominent light absorption properties. And KFs–MXene aerogel has an evaporation efficiency of 90.4% and evaporation rate of 1.47 kg m^−2^ h^−1^ at 1 sun. And other biomass nanofibers, including cellulose and CS, were utilized to modify the components of MXene aerogels and build their unique structures and exquisite wettability to increase the evaporation efficiency and rate [107,108].

**Figure 7:**
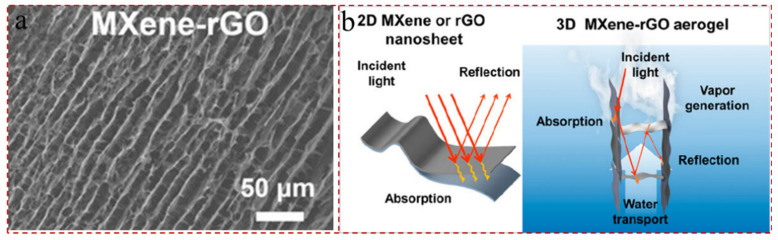
(**a**) SEM images of MXene-rGO hybrid aerogels with the directional freezing [113]. (**b**) Illustration of the solar energy harvesting for the MXene and MXene-rGO aerogels [113].

The 2D nanomaterials mainly include graphene, layered double hydroxides (LDHs), MoS_2_, hydrogen-bonded organic frameworks (HOFs), covalent–organic frameworks (COFs), metal–organic frameworks (MOFs), etc. [109]. 2D graphene-based materials have large specific surface area, superior hydrophilicity and intriguing photothermal conversion capacity and are served as solar evaporators [110,111]. Zhang et al. [112] successfully synthesized novel photothermal conversion materials, MXene/graphene aerogels (MXene/GAs), via a combined hydrothermal and freeze-drying process. The MXene dispersions were prepared by HF etching and tetramethylammonium hydroxide aqueous solution immersing, and the MXene/GAs possessed porous laminated structures and superior hydrophilicity (WCA of 0°). The evaporation rate of MXene/GAs was as high as 1.423 kg m^−2^ h^−1^, and the conversion efficiency could attain 89.6% at 1 sun. Similar in structure to graphene, the increased oxygen-containing functional group makes rGO more reactive, and rGO and MXene are more easily composited together due to the rigid chemical bonds. Wu et al. [113] prepared MXene suspensions using LiF and HCl etching, subsequently mixed with GO suspension to acquire MXene–rGO hybrid aerogels, and rGO nanosheets were adhered to the MXene framework. As displayed in Figure 7a,b, the MXene–rGO hybrid aerogels delivered excellent hydrophilicity (WCA of 0°) and vertically aligned channel architectures, which can strengthen the water transport and effectively lower the light reflection loss. The water evaporation rate and energy transfer efficiency of MXene–rGO hybrid aerogel was 2.84 kg m^−2^ h^−1^ and 96% under 1 sun, respectively. Due its low cost, simple preparation, large specific surface area, and adjustable aperture, transition metal sulfides (TMSs) are widely used in desalination field. MoS_2_, as the typical TMS, has emerged in interfacial evaporators, due to the commendable micro-surface chemistry, tunable microstructure, large specific surface area, and good light absorption. Yang et al. [114] synthesized MXene suspensions by HF etching, and the MXene/MoS_2_ aerogels were manufactured by regulating the MoS_2_ and MXene contents with chitosan (CS) crosslinking agent. The robust electrostatic attraction between CS and MoS_2_ enhanced the mechanical property of the MXene/CS aerogels. The narrow bandgap of MoS_2_ and the hydrophilic and vertical pore structure of MXene conferred the MXene/MoS_2_ aerogel a seawater evaporation rate of 2.78 kg m^−2^ h^−1^.

Compared with traditional 2D structures (such as sheet, membrane, and layered structures), 3D materials have unique merits, including a large evaporation area and low heat loss. In particular, biomimetic structure evaporators are attracting increasing attention due to their low energy consumption, high efficiency, and minimal environmental impact [115,116]. The multilayered 3D interconnected structure has attracted pervasive attention from researchers due to its distinguishing traits. Unlike point-to-point contact between particles or 1D nanowires, it can provide interconnected and convenient channels for water vapor overflow; establish longitudinal backflow and lateral, parallel diffusion paths for salt ions; and impart excellent mechanical strength to the evaporator. For example, wood, as a typical 3D biomass material, is low-cost, non-toxic, and degradable, and it has a high mechanical strength. In particular, wood has promising potential as a substrate for interfacial evaporation due to its natural hydrophilicity, complex microstructure (microchannels with mesoporous, hierarchical structures, and low curvature), and extremely low thermal conductivity (thermal localization) [117,118]. Gao et al. [119] described a sandwich-structured MXene/wood aerogel coupled with phase change materials for evaporators, in which flexible, mold-resistant delignified natural wood aerogel (DWA) was used as the substrate for the interfacial evaporator, and layered MXene was attached to the porous surface of the substrate as the photothermal layer. The obtained evaporator exhibited satisfying thermal insulation and incremental photothermal response, and the evaporation rate was up to 2.0 kg m^−2^ h^−1^ under 1 sun.

#### 3.2.2. MXene-Based Hydrophobic Aerogels

**Figure 8:**
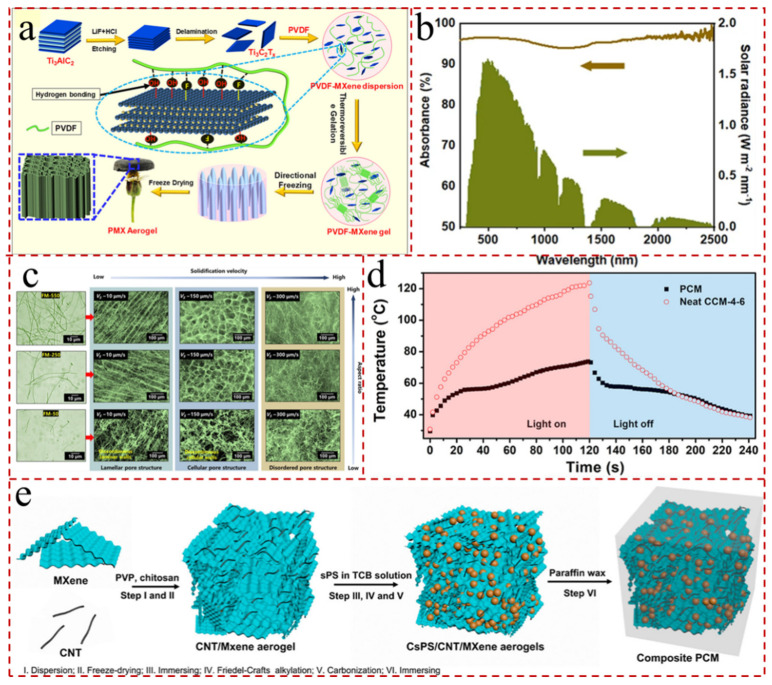
(**a**) Schematic illustration of the preparation of thermoreversible gels of PVDF/MXene composites, unidirectional freezing, and the preparation of PVDF/MXene hybrid aerogels [123]. (**b**) Solar spectrum (AM 1.5) and UV-vis absorbance of l-FMAs in the range of 200–2500 nm (wet state) [128]. (**c**) FM aerogels with tunable pore structures produced via regulation of aspect ratio and solidification velocity [128]. (**d**) Time–temperature curves of PCM-4-6 and CCM-4-6 under 1 sun [78]. (**e**) Schematic fabrication of the CsPS/CNT/MXene aerogels and composite PCMs [78].

Although MXene-based hydrophilic aerogels can facilitate water transport and promote evaporative escape, the precipitated salts can deposit on the surface of the evaporators during the continuous desalination. This phenomenon not only reduces the light absorption capacity, but it also blocks water vapor transmission over time due to the relatively small pore sizes inside the aerogels, therefore causing the reduced evaporation efficiency and rate [21,120]. The hydrophobic treatment of the aerogels can impede liquid water from penetrating inside the aerogels, thereby maintaining the dimensional stability of the aerogel architectures and preventing salt accumulation, resulting in a stable and efficient long-term desalination performance. Inspired by phenomena in nature comprising lotus leaves, butterfly wings, nepenthes, springtails, rose petals, salvinia, etc., the hydrophobic aerogels have two typical features, low surface free energy and rough surface structure [121,122]. Suresh et al. [123] utilized unidirectional freezing technical to successfully designed PVDF/MXene hybrid aerogels with anisotropic porous structures, and the acquired PVDF/MXene hybrid aerogels had good hydrophobicity (WCA of 153°; Figure 8a). The PVDF/MXene hybrid aerogels showed final temperatures of 55 °C after 10 min of residence at 140 °C. Bentonite possesses typical clay-like layered structures and features advantages such as a large surface area, excellent mechanical properties, and excellent thermal stability. The introduction of bentonite into MXene-based aerogel can further dramatically prohibit the oxidation of MXene. Tang et al. [124] constructed porous carboxylated cellulose nanofibers/defoliated bentonite/MXene (CNF-C/BTex/MXene) aerogels by simple in situ co-precipitation coupled with microwave hydrothermal and solution impregnation technique. The CNF-C/BTex/MXene aerogels displayed outstanding stability and favorable hydrophobicity (WCA of 149°), and the surface temperature of the harvested hybrid aerogels can reach 75.2 °C within 15 s under 1 sun.

The merits of hydrophobic hydrogels in SIED comprise (1) self-floating on water surfaces without additional support; and (2) hydrophobic surfaces, preventing salt fouling on photothermal membrane surfaces. However, hydrophobic hydrogels, as a physical barrier to heat and mass transfer, also present challenges. Heat transfer from the photothermal conversion interface (top surface) to the evaporation interface (bottom surface) is constrained by the porous structure-trapped air within, forming an insulating layer [125,126]. Furthermore, the porous structure increases vapor transmission resistance, while the effective evaporation area is also influenced by the membrane’s porosity. Moreover, photothermal molecularly driven hydrogels require hydrophobicity to prevent pore wetting, whilst superhydrophobic surfaces resist wetting-induced fouling and scaling.

Pore structure control is particularly important in the microscopic morphology regulation of evaporators. The abundant pores and rough surfaces not only give the evaporator superb hydrophobicity; they also lessen the upward reflection of sunlight, so that sunlight is absorbed through multiple scattering [83,127]. In general, the macroporous structures are more conducive to water transport and vapor escape. On the contrary, smaller diameters, especially nanoscale pores that do not exceed the average free range of air, have better heat preservation. The 3D fibrous aerogels with tunable pore structures (lamellar pores, cellular pores, and disordered pores) were constructed by varying the aspect ratio and solidification rate of fibrous MXene (FM) via ice crystal-assisted assembly [128] (Figure 8c). Among three aerogels, the FMs aerogels with lamellar pores delivered superior light absorbance above 93.5% in the entire solar spectrum and could attain an average evaporation rate as high as 1.482 kg m^−2^ h^−1^, with a light-to-heat conversion efficiency up to 92.08% (Figure 8b). Furthermore, Gui et al. [78] engineered novel carbonized syndiotactic polystyrene/CNT/MXene (CsPS/CNT/MXene) aerogels with distinctive egg-box structures containing numerous nanofibrous carbon microspheres within the framework (Figure 8d). The maximum WCA of various CsPS/CNT/MXene aerogels exceeded 148.2°, and the temperature of the hybrid aerogels increased steadily until attaining 123.7 °C at 120s, while the heat generated by solar radiation was balanced by the heat dissipated by the radiative heat flux (Figure 8e).

#### 3.2.3. MXene-Based Janus Aerogels

**Figure 9:**
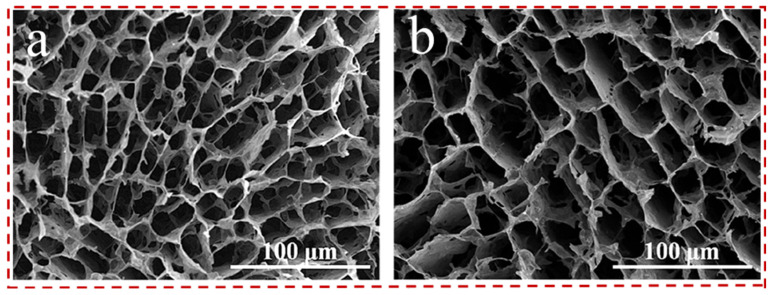
SEM images of the Janus VA-MXA: (**a**) upper and (**b**) bottom layers [134].

To date, it is widely known that the evaporation rate is associated with the phase change of water and the subsequent diffusion of water vapor. In order to accelerate the phase transition, Janus aerogels have been recognized as optimal materials due to their distinctive asymmetric wettability [129,130]. The conventional Janus aerogel evaporators consist of a hydrophobic photothermal layer on one side and a hydrophilic water supply layer on the other. The hydrophobic photothermal surface is a light-absorbing layer that converts the absorbed light energy into thermal energy. Then, the heat energy is conducted to the interfaces of the hydrophilic and hydrophobic layers to emerge as water vapor [131,132]. This construction minimizes heat loss and guarantees adequate water supply to promote efficient water vapor generation, thereby accomplishing excellent evaporation performance [133]. In addition, the ingenious architecture can inhibit the hydrophobic layers above from precipitating, and the balance between high evaporation and salt tolerance is addressed through continuous water delivery and rapid dissolution of salt crystals. For instance, Zhang et al. [134] elaborately designed an MXene aerogel (MXA) with vertically aligned channels and then modified the VA-MXA with fluorinated alkyl silane to obtain the hydrophobic upper layer (Figure 9a,b). Some features, such as superior internal light-to-heat conversion efficiency, rapid water diffusion, strong capillary pumping, etc., enabled the Janus VA-MXA to achieve a high and stable solar steam production rate of 1.46 kg m^−2^ h^−1^ within 15 days, with an energy conversion efficiency of approximately 87%. And under sunlight, the hydrophobic upper layer collected solar energy and converted the light into heat, and then it heated only a small portion of the seawater in contact with the hydrophobic layer to generate vapor that escaped along vertical channels. In addition, the thermal confinement effect at the hydrophobic–hydrophilic interface can effectively concentrate the heat and encourage in situ evaporation of water from the hydrophilic bottom layer. Due to high specific surface area, renewability, and biodegradability, cellulose nanofibers (CNFs) have attracted widespread attention [135,136]. And the abundant functional groups make CNFs easy to modify and combine with other functional materials. Han et al. [137] manufactured novel Janus CNF/MXene composite aerogels as interfacial evaporators by pre-freezing, solvent exchange, unidirectional freeze casting, and freeze-drying. One side of CNF/MXene aerogel was modified by silane to form a hydrophobic layer and acted as heat insulation for efficient evaporation of water. The upper surface temperature of Janus JCNF/MXene aerogel under 1, 2, and 3 suns was 46.0, 77.2, and 92.5 °C with the steady-state evaporation rate of 2.16, 3.20, and 3.93 kg m^−2^ h^−1^, respectively. Loofah sponges were connected by fiber skeletons, establishing a permeable 3D highly porous network with excellent mechanical properties and high hydrophilicity [138]. Wang et al. [139] designed a 3D Janus MXene/CNFs/luffa (JMCL) aerogel with excellent mechanical properties for a biodegradable interfacial solar steam generation device. The Janus texture empowered the JMCL aerogel continuous water transportation and high salt rejection. The average surface temperature could attain 30.8 °C and 45.2 °C under 0.5 sun and 2 suns, respectively. The concentration values of Na^+^, K^+^, Mg^2+^, and Ca^2+^ over desalination decreased drastically to 2.25, 0.28, 1.02, and 0.35 mg L^−1^, respectively, which were obviously below those of the World Health Organization.

Despite the pronounced advantages of MXene-based Janus aerogels, which can even float autonomously in solutions, they occasionally require suspension or support from an insulating layer to minimize heat loss during evaporation. To reduce vapor transmission resistance and enhance interfacial heating efficiency, a thin hydrophobic layer is typically employed. However, when this hydrophobic layer is excessively thin, salt deposition may occur on its hydrophobic surface (Table 1).

## 4. MXene-Based Hydrogel

### 4.1. Design of Hydrogel Materials

Hydrogels, as 3D crosslinked polymeric materials, are generally developed from polymers with attractive stability through corresponding physical and chemical crosslinking [140,141]. Hydrogels have received widespread attention as carrier materials for interfacial solar water purification, because the 3D gradient internal architectures (e.g., internal interstices, micron avenues, molecular networks, etc.) can dramatically facilitate water transport and diffusion, the administrable molecular network texture can effectively coordinate the water molecule contents in various states (e.g., bound, intermediate, and free water), and the regulation of structure engineering can further enhance the evaporation rate and efficiency [142,143]. Therefore, it is possible to effectively regulate the water content in disparate states by managing the crosslinking density and molecular network frameworks, thereby diminishing the enthalpy change of water vaporization in hydrogels. In addition, the introduction of light absorbers (e.g., organic fillers and inorganic functional materials) to hydrogels can have an effect on the enthalpy of vaporization of the hydrogels, which is mainly attributed to the disrupted hydrogel [144,145].

The structure of hydrogels is generally constituted by physical (non-covalent) or chemical (covalent) crosslinking. Chemical crosslinking mainly includes free radical polymerization, borate bonding, condensation reactions, aldehyde crosslinking, click chemistry, etc. [146,147,148]. Physically crosslinked hydrogels are mainly composed of hydrogen bonding, ionic bonding, metal coordination, van der Waals forces, chain entanglement, hydrophobic bonding, π-π stacking, etc. [149,150]. It is noteworthy that the mechanical properties and flexibility of hydrogels are engineered by intelligently modulating physical or chemical crosslinking cooperations.

#### 4.1.1. Chemically Crosslinked Hydrogels

Chemically crosslinked hydrogels are synthesized by chain growth polymerization, addition and coalescence polymerization, and electron beam engagement [151,152]. Among them, the chain growth polymerization mainly encompasses the reactions of free radicals, controlled free radicals, anions, and cations, which are completed by three courses, namely initiation, propagation, and termination [153,154]. Subsequently, the generated radical active sites react with monomers in a chain-like manner, which assures the mechanical properties and stability of the hydrogels. In practical, the outstanding mechanical properties grant hydrogels, as solar-powered interfacial evaporators, prominent durability and a prominent lifespan [155,156,157]. Among multitude chemical crosslinking reactions, the free radical polymerization plays an important role in the construction of hydrogels. Due to the high chemical activity of the free radicals, hydrogels can be rapidly generated in the presence of initiators and crosslinking agents. For example, Zou et al. [158] successfully grafted polyethyleneimine (PEI) onto MXene surfaces to obtain the poly (acrylamide-co-acrylic acid) nanocomposite hydrogel with MX-PEIS nanosheets (MX-PEIS/Gel) via tannic acid-mediated self-assembly polymerization through Schiff-base or Michael addition reaction. The acquired X-PEIS featured considerable ameliorations not only in anti-fouling performance but also in mechanical properties, expansion characteristics, water retention capacity, and lubricity. Xu et al. [159] successfully fabricated 3D hydrogel foams with dual-network architecture via a controlled foaming–gelation technique. The guluronic acid remaining on the G block between the two sodium alginate backbones spontaneously binds Ca^2+^, using CaCO_3_ as a releasing agent, to establish the crosslinked network of sodium alginate. Meanwhile, under the same acidic conditions, the aldehyde group on glutaraldehyde reacts with the hydroxyl group on the PVA chain to form the second crosslinked network through covalent bonds. As a result, the obtained hydrogel foam can attain a high water evaporation rate of 2.12 kg m^−2^ h^−1^ under 1 sun. In order to facilitate the strength, durability, and lifespan of the hydrogel evaporators, it is generally essential to introduce reactive groups, initiators, and catalysts into the polymer matrix for polymerization. Moreover, the crosslink density, as one of the important parameters of mechanical properties, can be regulated by varying the concentration of irradiation intensity, crosslinking agent, concentration of photoinitiator, and reaction time.

#### 4.1.2. Physically Crosslinked Hydrogels

Physically crosslinked hydrogels are constituted when crosslinking occurs via ionic or intermolecular interactions (e.g., ionic bonding, dipole–dipole, dipole-induced, metal–ligand coordination, hydrogen bonding, and hydrophobic associations) [160,161]. Compared to chemical crosslinking, physically crosslinked hydrogels can be reversible, and the self-healing and degradation behaviors of the hydrogels can be effectively authorized by modulating conditions such as pH, temperature, or ionic interactions [162,163]. In general, the preparation process of physical crosslinked hydrogels does not involve toxic crosslinking agents, thus guaranteeing green, non-polluting, and environmentally friendly features and characteristics [164,165]. The manufacture course of physical hydrogels generally comprises freeze–thaw cycles, phase-inversion, heating and cooling, pH conversion, mixing polycations and polyanions, or mixing polyelectrolyte solutions with oppositely charged multivalent ions [166].

Ionic interactions, driven by the mutual attraction of opposite electric charges, can crosslink a network. In detail, the polyanionic polymer can be crosslinked with cations, while the polycationic polymer may be interacted with anions [167,168]. Polymers are capable of reacting with one or more opposing charges to form polyelectrolyte complexes, for example, sodium alginate–Ca^2+^, CS–glycerol phosphate, alginate–CS, collagen–CS, chondroitin–CS sulfate, etc. The ion-crosslinked hydrogels possess the advantages of simple preparation condition without covalent modification, but it is difficult to control gelation rate, which can bring about inhomogeneous precipitation [169,170]. Thus, delayed gelation inhibition buffer has been introduced into the hydrogels to control the gelation kinetics. Elmehbad et al. [171] successfully prepared two novel chemically crosslinked CS hydrogels by inserting oxalyl dihydrazide groups (oxalyl dihydrazide as delayed gelation inhibition buffer) between CS Schiff-base chains and CS chains. Another challenge for ionic gelling is its unpredictable long-term stability in vivo because of the rapid rate of ion exchange and gel dissolution in physiological medium.

Metal–ligand coordination is fully crosslinked by ligand-modified complexes between polymers and metal ions. The mechanical properties of the metal-coordinated hydrogels are associated with the density and dynamics of metal–ligand interactions [172]. In general, telechelic metal-coordinated hydrogels encompass catechol or histidine ligands, but hydrogels have been reported to incorporate the stronger complexing agents (nitrocatechol) [173,174]. For example, Miao et al. [175] synthesized composite hydrogels via a double-crosslinked hydrogel strategy including copolymerization of acrylic acid (AA) and acrylamide (AM) and subsequent coordination between copolymer P(AA-AM) and Co^2+^. Inspired by the self-assembling metal-reinforced mussel holdfast threads, Kim et al. [176] employed a hydrogel consisting of catechol-functionalized four-arm polyethylene glycol polymers that were crosslinked in situ via permanent catechol–catechol covalent bonds and transient Fe^3+^–catechol metal-coordinate complexes.

The electrostatic repulsive interactions between MXene nanosheets and their favorable hydrophilicity permit uniform dispersion of MXene nanosheets within the hydrogel matrix. Moreover, polymer chains become tightly entangled with these MXene nanosheets via hydrogen bonds between MXene surface functional groups (e.g., -F, -O, and -OH) and -OH groups of PVA, forming a secondary crosslinking network that further prevents autocrosslinking [177]. As a gelling agent, co-gelling agent, and nanofiller, MXene can enhance hydrogel’s various properties and endow it with surprisingly novel functionalities [178]. For instance, it was found that dispersions formed from single or multiple stacked MXene nanosheets, at identical concentrations, exhibit distinguishing elastic and viscous moduli, which would reflect on the rheological properties of the hydrogel [179]. And Li et al. [180] facilitated super-tough and highly stretchable physical hydrogels that are conductive on multibond network/MXene nanosheets. The MXene–polyacrylic acid–Fe^3+^ magnetic nanocrystal physical hydrogel could be achieved by controlling Fe^3+^ permeation and exhibited brilliant and balanced mechanical properties.

### 4.2. Functional MXene-Based Hydrogels

**Figure 10:**
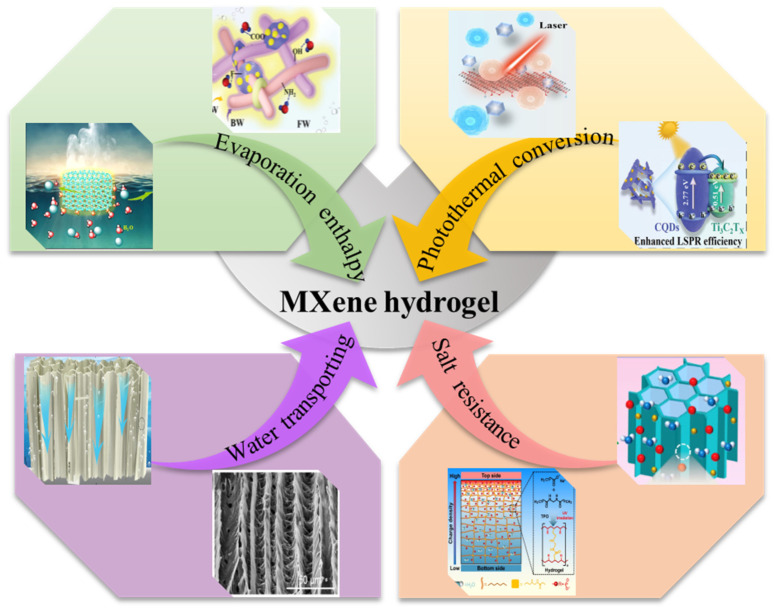
Rational design of functional MXene-based hydrogels.

MXene-based hydrogels, as ideal solar evaporators, are capable of achieving excellent evaporation efficiency and long-term stability. The approach for establishing efficient solar-powered interfacial evaporators mainly comprises the following essential components: (i) possessing a high-efficiency photothermal conversion capacity to improve solar energy utilization, (ii) optimizing the thermal management system to reduce the heat conduction loss, (iii) designing convenient pore architectures to provide an avenue for the water transport, and (iv) taking into account salt resistance to guarantee long-term stability in seawater (Figure 10) [181,182,183]. By regulating and optimizing the structural and functional parameters, the solar-powered interfacial evaporator is able to fulfill water transport from the bottom to the surface of the evaporator, prompt water evaporation, and achieve high energy conversion efficiency [184,185].

#### 4.2.1. Photothermal Conversion Capacity of MXene-Based Hydrogel

The rapid development of solar-driven interfacial evaporators is inseparable from the contribution of the synthesis and structural design for photothermal materials. The high-efficiency capability to harvest light is an important prerequisite for materials to convert light into thermal energy. Therefore, the photothermal materials must fulfill the following criteria: a wide range of light absorption and a high light absorption rate. This also implies that they have low transmittance and delicate reflectance of light across the entire solar spectrum (200–2500 nm) [186,187]. The photothermal materials can convert the captured solar energy into various typological energy, such as heat, electricity, and chemical energy. Among them, the photothermal conversion is a direct conversion process with the highest energy efficiency [188,189]. And the mechanisms of interaction between electromagnetic radiation and photothermal materials can be summarized into three categories: LSPR effects, electron–hole generation and relaxation, and conjugate or super conjugation effects [190,191].

**Figure 11:**
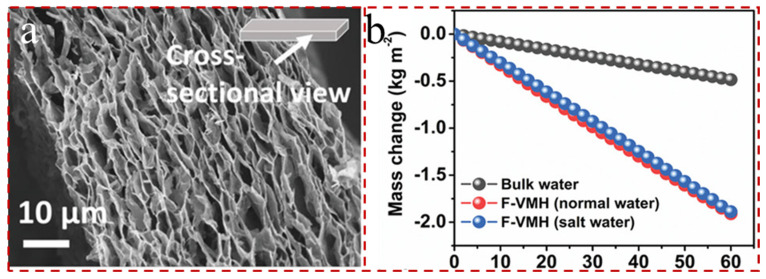
(**a**) Cross-sectional FESEM image of MXene 2D hydrogel, showing its porous assembly [200]. (**b**) Mass change of water, indicating evaporation rates with normal and simulated seawater compared with bulk water evaporation under 1 sun irradiation [200].

MXene has the ability for electron–hole generation and relaxation, and it is able to cover the plasmon peaks in the entire visible and near-infrared spectra. When the incident photon energy exceeds the bandgap energy, the MXene is able to excite the transition to produce electron–hole pairs [192,193]. Then, the electron–hole pairs relax to the edge of the energy band, and the energy is released through Auger recombination, radiation relaxation in the photon form, or non-radiative relaxation in the phonon form [194,195]. When energy is released in the form of phonons, it can provoke the lattice into heating to convert energy into heat. Additionally, the inter-band leaps give MXene a strong absorption ability in the ultraviolet range, making it able to achieve a very high efficiency of photothermal conversion [196,197,198]. Therefore, MXene has good prospects as a candidate for photothermal conversion materials. In particular, MXene has the theoretical ability to convert approximately 100% of incident light into heat, which ensures that it is well suited for photothermal evaporation [199]. Dutta et al. [200] developed field-induced vertically aligned MXene hydrogels (F-VMHs) via an electric field-assisted forced-assembly technique (Figure 11a). The evaporation rate of the F-VMH was measured to be 1.91 kg m^−2^ h^−1^ with fresh water, almost fourfold higher than the corresponding bulk water evaporation (0.48 kg m^−2^ h^−1^) under 1 sun (Figure 11b).

Due to poor mechanical properties and relatively low steam generation rates, various functional materials have been introduced into the substrate to address these puzzles [201,202]. The hydrophilic groups in the hydrogel network could hasten the water evaporation rate [203,204]. The rGO framework retains abundant oxygen-containing functional groups and favors the reduction of the enthalpy of evaporation of water. Li et al. fabricated rGO/MXene (A-rGO/MXene) hybrid hydrogels with vertically aligned structures as the stand-alone solar vapor generation devices. The A-RGO/MX hydrogel achieved an outstanding water evaporation rate of 2.09 kg m^−2^ h^−1^, with a high conversion efficiency of 93.5% under 1 sun, and removed more than 99% of most ions (Na^+^, K^+^, Ca^2+^, Mg^2+^, and B^3+^) in seawater.

In view of the fact that solar evaporators are applied in polluted water bodies, their antimicrobial properties have great potential. Metallic Ag is an intriguing candidate for incremental antimicrobial properties as a result of its ability to release silver ions [81,205,206]. And metallic Ag is also regarded as a special light absorber due to its unique LSPR effect, especially within the visible spectral range [207,208]. Furthermore, inserting Ag between MXene layers can avert layer stacking and improve the mechanical properties of the evaporators. Zhu et al. [209] manufactured organic–inorganic composite hydrogels consisting of double-network polyacrylamide/sodium alginate (PAM/SA), MXene@Ag (MA) nanosheets, and inorganic hydrated salt (Na_2_SO_4_·10H_2_O, SSD) by the one-pot polymerization method. The composite hydrogels presented a heating plateau between 33.9 °C and 38.6 °C, and a cooling plateau between 24.8 °C and 26.7 °C. Compared with PAM/SA/SSD hydrogel, the antibacterial radius of the composite hydrogel was increased by 24%, and the antibacterial efficiency was incremented by 56% after 5 min of near-infrared irradiation at 808 nm, with an intensity of 3 W/cm.

#### 4.2.2. Water Transporting of MXene-Based Hydrogel

The continuous water supply makes a huge difference to the photoconversion efficiency and vapor condensation. It is essential to precisely match the water supply rate with the steam generation rate. Delivering excessive water can result in increased heat conduction loss, while grossly inadequate water is not sufficient to produce vapor, bringing about the accumulation of salt on the surface of the evaporators and conversely affecting the light absorption performance and the steam generation of the system [24,210]. However, the phenomenon of stacking or agglomeration of MXene nanosheets leads to poor water transfer and vapor overflow of conventional MXene-based hydrogels during the evaporation process [63,211]. The porous structures, vertically aligned (oriented) channels, core–shell architectures, hydrophilic surfaces, and large layer spacing, as well as the use of capillary forces to transport water from the bottom to the top, can tackle such conundrums by providing sufficient water for evaporation from the top [212]. The interior of MXene-based hydrogel interface evaporators features a rich pore network, and the optimized pore architecture ensures efficient water transport. Internal gel structures are typically modified by adjusting pore distribution and aperture, employing primary techniques, including ice-molding, directional freezing, and fermentation technology. Inspired by natural wood and cuttlebone, Chen et al. [213] developed cuttlebone-templated directional porous hydrogel (CTDPH), which was composed of MXene, tetramethylenediamine, and ammonium persulfate crosslinked by polyethylene glycol diacrylate by etching sea bone in the hydrogel network. The CTDPH has a penetrating oriented macroporous structure, enabling rapid seawater transport while effectively preventing salt crystallization on the surface of the hydrogel. The CTDPH can largely increase the seawater evaporation rate from 0.73 to 3.11 kg m^−2^ h^−1^ under 1 sun, and it still maintained 91.6% of its original solar evaporation rate (2.85 kg m^−2^ h^−1^) even after undergoing 10 cycles of seawater evaporation.

**Figure 12:**
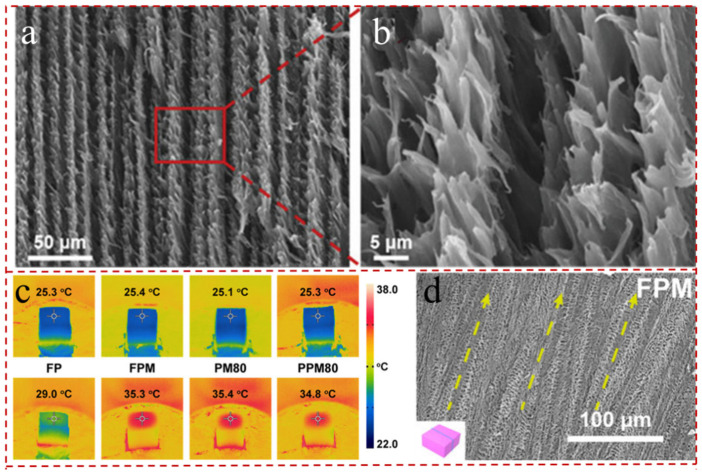
(**a**,**b**) SEM images of the cross-section view of tree-inspired hydrogel-10%PVA with different magnifications (red square and dashed line: local magnification) [214]. (**c**) SEM images of the vertical section for FPM [77]. (**d**) Schematic illustration of the preparation process of tree-inspired hydrogel (yellow dashed arrow: vertical section) [77].

The vertically aligned channels in the aerogel evaporator can effectively diminish heat loss and propel water transport at the gas–liquid interfaces, thereby improving evaporation efficiency and facilitating steam condensation. Hu et al. [77] fabricated anisotropic PVA/MXene composite hydrogels with the vertically oriented internal channels (Figure 12c). The optimal PVA/MXene composite hydrogel evaporator achieved a superior water evaporation rate and a high energy efficiency, as well as showing high surface temperature due to surface wettability modification and unique structures. Meanwhile, inspired by water transport in microchannels inside trees, Yu et al. [214] prepared PVA-GO–MXene hydrogels with vertically aligned channels, and the hydrogels can reduce the energy requirement for water evaporation by modulating the interaction between water molecules and the hydrophilic polymer network and manifest excellent internal photothermal conversion efficiency (Figure 12a,b,d).

The core–shell structures can significantly enhance the toughness of hydrogels by 3D physical and/or chemical crosslinkages. The shell layer of the skin–core structure generally employs hydrogel with a small pore size and eminent water permeability, which can rapidly and directionally transport the obtained water to the inner layer at night, obstructing the water evaporation [215,216,217]. And the core hydrogel possesses large pore dimensions and intensive water release capability, which could effortlessly compile absorbed water at night. Overall, the synergistic hygroscopic effect between the core–shell layers can significantly improve the core–shell hydrogel’s efficiency in regard to water collection and release [218,219]. Pi et al. [220] designed a MXene/PDA hydrogel with a core–shell structure, in which hydrophobic associative crosslinked polyacrylamide, as the core layer, absorbed and transported water, and PVA hydrogel, as the shell layer, performed interfacial solar evaporation. The obtained core–shell structure hydrogel solar evaporator has prominent mechanical properties and an energy efficiency of 94.7% under 1 sun, and the temperature of the composite hydrogel reached 38.7 °C over 20 min under 1 sun.

#### 4.2.3. Salt Resistance of MXene-Based Hydrogel

Salt can accumulate on the surface of the evaporator during the steam generation process. Driven by the concentration gradient, salt ions diffuse down the channel mainly through advection and diffusion and salt deposition occurs when the salt accumulation rate exceeds the diffusion rate [221,222]. The increased salt not only reduces the harvest of visible light from the photothermal materials, but also hinders the gas–liquid circulation, causing diminished evaporation rate and efficiency or even complete destruction of the evaporation performance [31,223]. The Marangoni effect is employed to address salt retention, a phenomenon that utilizes surface tension differences (such as temperature or pressure gradients) to induce fluid flow from regions of higher surface tension towards those of lower surface tension [224,225]. This constitutes a long-term strategy for effective retention of effluent salt. The Marangoni effect enables salt retention in MXene-based hydrogel interface evaporators through the construction of dual-mode exchange channels [226,227,228].

Typically, the conundrum is settled by repeated washing, the construction of large vertical pores, the design of Janus structures, etc. Porphyrin, as a multifunctional crosslinker, is characterized by conjugated π-π cooperations and ring-shaped distribution of -COOH groups and can significantly promote the evolution of hydrophilic networks. Zhang et al. [229] exploited the existence of charge redistribution and coupling interaction between MXene and porphyrin and fabricated MXene/PVA/porphyrin hydrogel membranes. The oriented self-stacking of nanosheets and the special hydrophilic network structure gave the hydrogel membrane a stable water evaporation rate of 1.72 ± 0.4 kg m^−2^ h^−1^ in real seawater. This was associated with an efficient capillarity force, osmotic expansion, and transpiration effect. These processes allow the concentrated salt to diffuse back down into the bulk water and prevent salt precipitation.

**Figure 13:**
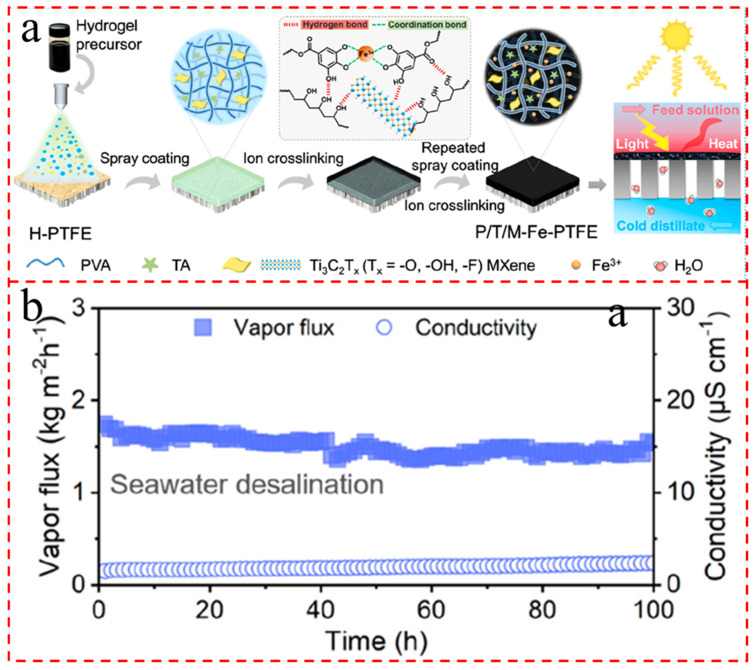
(**a**) Schematic illustration of the sequential spray-coating and ion-crosslinking procedures for fabricating a P/T/M-Fe-PTFE membrane [232]. (**b**) Long-term stability of the P/T/M-Fe-PTFE membrane during seawater desalination under 1 sun [232].

Due to stable metal–ligand coordination bonds and multiple network structures, various metal ions are employed for crosslinking hydrogels and improve their mechanical properties [230,231]. Given the stable tannic acid (TA)–Fe^3+^ network architecture and robust Fe^3+^-PVA network structure, the PVA/TA/MXene (P/T/M) hydrogel endowed the P/T/M-Fe-PTFE composite membrane with excellent surface self-heating, self-cleaning, and fouling-rejection capabilities (Figure 13a) [232]. The obtained P/T/M-Fe-PTFE composite membrane manifested a prominent freshwater production rate (1.71 kg m^−2^ h^−1^), high ion rejections (e.g., boron rejection of 99.24%), and forceful durability when desalinating real seawater for 100 h without flux decline (Figure 13b). Kapok fibers possess a high porosity and hole structure, with dimensions much larger than the free path of water vapor. As evaporators, the inherent large pores of kapok fibers can minimize molecular collisions, thus thoroughly reducing the thermal conductivity of water vapor [233]. Meanwhile, these large pores also lessen collisions between water vapor molecules and the backbone [234]. Su et al. [235] developed a 3D sandwich hydrogel (MXene-decorated kapok fibers/PVA hydrogel) with an asymmetric structure as solar-driven interfacial evaporation. The top layer served as the light-absorbing layer, the middle layer was MXene-decorated kapok fibers homogeneously dispersed in the composite hydrogel, and the bottom layer with the oriented structure view functioned as the water delivery channel. The evaporation rates of the evaporator fluctuated around 2.12 kg m^−2^ h^−1^ over 12 cycles and were 1.71, 1.61, and 1.44 kg m^−2^ h^−1^ over 6 h in solutions with NaCl concentrations of 10, 15, and 20 wt%, respectively.

#### 4.2.4. Reduction of Evaporation Enthalpy of MXene-Based Hydrogel

The harvested solar energy is not only converted to heat for steam generation; it is also dissipated into the underlying water bodies and the surrounding environment through heat conduction(*q_cond_*), heat convection(*q_conv_*), and heat radiation (*q_rad_*) [236,237]. In order to obtain a high evaporation efficiency and rate, it is inevitable to implement effective thermal management for the system.

The enthalpy of evaporation in interfacial evaporation is the amount of heat absorbed when water is transformed from the liquid to the gaseous state at the same temperature and pressure [238,239]. The heat, *Q*, required to evaporate a large quantity of water at constant external pressure is equal to the enthalpy of evaporation of bulk water [240].

(1) $${△}_{vap}H^{BW}=H^{V}-H^{L}$$

where *H^V^* is the enthalpy of water vapor, and *H^L^* stands for the enthalpy of bulk liquid water. It is indispensable to break the hydrogen bonds that are initially inherent in the water with the absorbed heat energy during the conversion of liquid water to vapor.

Water in hydrogels exists in the form of bound water (BW), free water (FW), and intermediate water (IW) [241]. Among them, water molecules in the IW state possess the lowest vaporization enthalpy [242,243]. It is an effective tactic to regulate the IW amounts in the hydrogel by varying the network density of the hydrogel. For example, Xing et al. [244] engineered the hierarchical pore-in-pore structure and porosity >95% of PVA/MXene/F-127 foam hydrogel using triblock copolymer (F-127) as a crosslinking agent. The PVA/MXene/F-127 foam hydrogels displayed more diminished the enthalpy of evaporation values than that of bulk water and implicated an evaporation rate of 4.1 ± 0.1 kg m^−2^ h^−1^, with an energy efficiency as high as 128.8 ± 2.0% under 1 sun. Inspired by the transpiration and natural photosynthesis of plants, Fan et al. [245] interpenetrated MXene and La_0.5_Sr_0.5_CoO_3_ (LSC) into a polymer network of PVA and CS to construct a well-designed photocatalyst-embedded photohydrothermal gel. Solar energy absorbed by MXene–LSC hydrogels was converted into water vapor (~92.30%), thermal convection (~1.40%), thermal radiation (~2.28%), and downward heat transfer (~0.80%). The average solar absorption was 94.7%, and the evaporation enthalpy of the optimum MXene–LSC hydrogel was 1175 J g^−1^, which was rather lower than pure water (~2250 J g^−1^). The MXene-based photothermal fabrics with various three-dimensional structures can not only provide excellent mechanical stability to the evaporators but also contribute effective and abundant channels for water supply and salt resistance during the evaporation process (Table 2) [246]. Han et al. [247] prepared robust MXene/PVA hydrogel-coated cotton fabrics (MHCFs), and their matrixes furnished layered porous channels for efficient water transport, causing a lower enthalpy (2090 J g^−1^) of water evaporation and outstanding efficiency (95%) of solar evaporation under 1 sun.

## 5. Summary and Prospects

In conclusion, in light of the expansion of cities and industries, the scarcity of freshwater resources has become a significant challenge to human health and long-term social development. The deployment of efficient and non-polluting SIED technology to produce clean water is one of the most promising strategies for addressing this issue. Currently, the evaporation rate of SIED systems has markedly increased from ∼1 to ∼4 kg m^−2^ h^−1^ under 1 sun, and their evaporation efficiencies have exceeded 80%, with many surpassing 90%. Photothermal materials, as the core of SIED systems, have emerged as a focal point in this field. Due to the excellent solar-thermal conversion and easily regulated surface chemistry, MXene is widely used in the desalination domain, and various MXene-based gels have performed eminent evaporation rates and efficiencies. Based on this, this paper focuses on the application progress of MXene-based aerogels/hydrogels in SIED over the past few years. Firstly, the preparation and principal properties of MXene are discussed. Then, the synthesis strategy and varying wettability of MXene-based aerogels are outlined and analyzed. Finally, for optimization and improvement, four major aspects of photothermal conversion capacity, namely water transport capacity, evaporation enthalpy regulation, and salt resistance, are combined with the excellent properties of MXene-based hydrogels.

**Figure 14:**
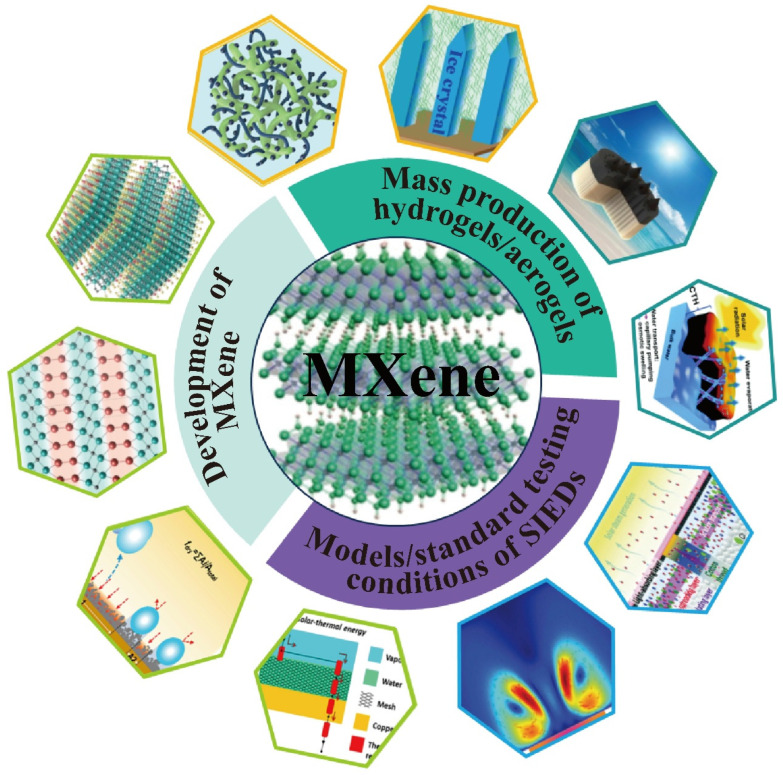
Conclusions and outlook for the MXene-based hydrogels/aerogels in solar-driven interfacial evaporation.

Despite the fact that SIEDs have displayed fascinating promise in recent years, MXene-based aerogels/hydrogels are still in the initial stages of SIED development, and there are a number of obstacles yet to be tackled (Figure 14).

(1) So far, there is yet no standard testing and characterization conditions, thus making it impossible to directly compare the evaporation efficiencies of various groups. Therefore, it is essential to develop the standards, which include measurement techniques and operating conditions of various SIED systems to guarantee fair comparisons.

(2) Currently, most research focuses on increasing the rate of photothermal conversion, designing water transport channels, reducing evaporation enthalpy, and enhancing salt resistance. There are relatively few explorations on high condensate collection rates. The freshwater productivity is positively correlated with the evaporation rate and the condensation collection rate. And the condensation collection technology is sluggish or even neglected, which seriously restrains the freshwater productivity.

(3) Most of the studies on SIED systems have been focused on the experimental components, while there are hardly any theoretical models for the deeper comprehension of the physical processes during evaporation. Surprisingly, it appears to be a simple process, but water evaporation in nanonetworks of aerogels/hydrogels is still not well understood.

(4) MXene is conventionally obtained through etching MAX using a top-down method, but the process can bring about harmful and toxic HF, which is hazardous to human health and pollutes the environment. Although less toxic chemicals (LiF/HCl, NaOH, and KOH) have been used to try to supplant HF, such research is still in the preliminary stages and requires extensive explorations.

(5) MXene with -O end groups exhibits optimal stability, attributable to the shielding of internal transition metal atoms from environmental exposure provided by the -O groups. However, -OH groups are prone to decomposition. Meanwhile, owing to their weaker chemical bonds, -F groups are often susceptible to desorption or substitution by other elements. Moreover, during layering and storage under ambient atmospheric conditions, individual MXene flakes readily oxidize into titanium oxide. This may compromise the long-term chemical stability of MXene, particularly in photothermal applications typically operating at elevated temperatures. To address this oxidation behavior, the antioxidant performance and reproducibility of MXene should be given high priority. Although several surface protection strategies have been reported to minimize or slow MXene oxidation, these effectively reduce MXene’s electrical conductivity levels. Consequently, enhancing the antioxidant properties and long-term stability of MXenes is imperative.

(6) The manufacturing process of most MXene-based aerogel interface evaporators is complex and costly, thus making it difficult to apply in practice, especially in some distressed and distant regions. The development of most MXene-based aerogel materials has been only limited to the laboratory, and many MXene-based aerogel devices are too miniaturized for large-scale applications. Therefore, MXene-based aerogels will move toward the direction of large-area, low-cost, and emerging technologies.

(7) There are potential adverse effects in the preparation of hydrogel materials, and synthesis methods need to be continually improved. The preparation process of MXene-based hydrogels is time-consuming and not conducive to large-scale production. The hydrogels, as solar-driven interfacial evaporators, suffer from decreased stability during long-term use. The self-healing MXene-based hydrogels are capable of healing themselves after repeated systematic damage, and they can be partially or fully restored to their original state within seconds or hours of the occurred damage. However, there have been few reports of self-healing MXene-based hydrogels in SIED systems that can withstand damage and achieve reliability and long-term stability.

(8) With the growing interest in aerogel applications in a variety of environmental domains, including water treatment, purification, and filtration, it is imperative to deliberate on its potential ramifications for human health and ecosystems. Whilst numerous natural materials possess inherent biocompatibility and biodegradability, due to their structural and degradation characteristics, other materials (like, polymers, MXene, etc.) may pose ecotoxicological risks. Although the prevailing consensus in the scientific community is that aerogels are generally considered safe materials, some researchers emphasize the need for further investigation into their potential specific risks. To date, standardization efforts concerning the ecotoxicity and cytotoxicity of aerogels remain incomplete. Furthermore, the preparation processes for aerogels and hydrogels have the potential to result in substantial carbon emissions, carbon dioxide, and solvent-recovery challenges.

## Figures and Tables

**Table 1:** MXene-based aerogels for highly efficient solar-driven interfacial evaporation (---: Not mentioned).

	Materials	Structural Characteristics	Functional Advantages	Evaporation Rate (kg m^−2^ h^−1^)	Efficiency (%)	Reference
Hydrophilic aerogels	PBAT/MXene aerogel	Hierarchical porous structure	Salt rejection	1.87	86.2	[21]
	MXene/aramid aerogel	Hierarchical pore structure	Capillary action	1.48	93.8	[104]
	Ag nanowires/MXene/aramid nanofibers	Porous network structure	Salt rejection, thermal insulation	2.21	92.0	[105]
	MXene-doped kapok fiber aerogel	Rich macroporous structure	Reduced heat loss	1.47	90.4	[106]
	MXene/graphene aerogel	Porous microstructure	---	1.42	89.6	[112]
	MXene–rGO hybrid aerogel	Vertically aligned channel structures	Reduced vaporization enthalpy	2.84	96.0	[113]
	MoS_2_–MXene/CS aerogel	Vertical pore channels	Salt rejection	2.78	---	[114]
	MXene/wood aerogel	Multi-stage water transport channel	Salt rejection, recover dissipated energy	1.77	92.6	[119]
Hydrophobic aerogels	MXene/RGO/carbon aerogel	Interconnected cellular architecture	Reduced vaporization enthalpy	1.48	89.2	[109]
	MXene aerogel	Ordered cellular/lamellar pore structures	Capillary action	1.48	92.1	[128]
	MXene aerogel	Well-ordered vertical array structure	Capillary water transport	1.46	87.0	[134]
Janus aerogels	Cellulose nanofibrils/MXene aerogel	---	Capillary action	2.28	88.2	[137]
	MXene-decorated CNFs/luffa aerogel	Dual-network architecture	Heat localization effect	1.40	91.0	[139]

**Table 2:** MXene-based hydrogels for highly efficient solar-driven interfacial evaporation. (---: Not mentioned).

Materials	Evaporation Rate (kg m^−2^ h^−1^)	Efficiency (%)	Note	Reference
MXene/PVA hydrogel	2.55	96.0	Water transporting	[77]
MXene hydrogel	1.91	94.5	Photothermal conversion capacity	[200]
PVA-GO–MXene hydrogel	2.71	90.7	Water transporting	[214]
MXene/carbonized wood hydrogel	3.71	129.6	Reduction of evaporation enthalpy	[216]
MXene/PDA/PVA-HAPAM hydrogel	3.02	94.7	Water transporting	[220]
MXene–PVA-MAP hydrogel	1.82	---	Salt resistance	[229]
PVA/TA/MXene hydrogel	1.71	---	Salt resistance	[232]
kapok fiber/MXene/PVA hydrogel	2.49	91.5	Salt resistance	[235]
PVA/MXene/F-127 hydrogel	4.10	---	Reduction of evaporation enthalpy	[244]
MXene/LSC/PVA/CS hydrogel	2.73	92.3	Reduction of evaporation enthalpy	[245]
rGO/MXene hybrid hydrogel	2.09	93.5	Photothermal conversion capacity	[248]

## Data Availability

Not applicable.
